# Culture Change and Eating Patterns: A Study of Georgian Women

**DOI:** 10.3389/fpsyt.2019.00619

**Published:** 2019-09-03

**Authors:** Ia Shekriladze, Nino Javakhishvili, Kate Tchanturia

**Affiliations:** ^1^D. Uznadze Institute of Psychology, School of Arts and Sciences, Ilia State University, Tbilisi, Georgia; ^2^Department of Psychological Medicine, Institute of Psychiatry, Psychology and Neuroscience, King’s College London, London, United Kingdom; ^3^Eating Disorders National Clinical Service, South London and Maudsley NHS Foundation Trust, London, United Kingdom

**Keywords:** acculturation, immigration, eating patterns, disordered eating, Eastern Europe, cross-culture, transition

## Abstract

**Introduction:** Immigration and culture change have been thought to affect various aspects of psychological well-being, including eating behaviors. This study aimed to examine the association between immigration, acculturation strategies and eating patterns.

**Materials and Methods:** Acculturation was conceptualized and measured by acculturation strategies of integration (maintaining original culture and adopting the new culture), assimilation (adopting the new culture and leaving behind the old), separation (sticking with the original culture only) and marginalization (maintaining/adopting neither culture). Eating patterns were conceptualized by dietary restriction, eating concern, shape concern, and weight concern. Links between demographic variables, acculturation strategies, and eating patterns were also examined. Five hundred and six Georgian women took part in the study: 253 living abroad (UK and USA) and 253 living in Georgia. Measures included East Asian Acculturation Measure (EAAM) for acculturation strategies (assimilation, integration, separation, and marginalization subscales) and Eating Disorder Examination Questionnaire (EDEQ) for eating patterns (dietary restriction, eating concern, weight concern, shape concern subscales, and global score). Relevant demographic variables and Body Mass Index (BMI) were recorded.

**Results:** Comparisons of immigrant and nonimmigrant groups using Multivariate Analysis of Covariance (MANCOVA) with BMI as a covariate found a difference in dietary restriction only, with immigrants yielding higher mean score than non-immigrants. The global EDEQ scores of immigrant and nonimmigrant groups were almost identical though. Correlations between separation and marginalization and four EDEQ scores were statistically significant and positive, while correlations between integration and two EDEQ subscales were marginally significant and negative. Regression analysis showed that separation and marginalization strategies of acculturation were significantly linked with EDEQ eating concern, shape concern, weight concern, and global scores thereby representing predictors of elevated eating outcomes.

**Discussion:** Findings suggested that moving to Western countries increased dietary restriction among Georgian women. Furthermore, while living abroad, the lack of integration in a host culture, as a common denominator of separation and marginalization strategies of acculturation, may predict elevated eating, shape, and weight concerns among women relocated over six years ago. Acculturation conditions may also be linked with integration or well-being outcomes.

## Introduction

Disordered eating patterns represent eating related unhealthy behaviors (e.g., excessive concern about weight and shape, excessive dieting, self-induced vomiting) that resemble Eating Disorders (ED) but are exhibited in a smaller degree in terms of frequency and intensity (1). Traditionally believed to be affecting women of affluent societies (2, 3), the prevalence of EDs has been increasing among diverse populations and cultures (4–8). While researchers are trying to find out more about the main determinants, risk and protective factors of EDs, it is recognized that they are culturally influenced (9–13). Among other factors, disordered eating has been linked with immigration, acculturative stress and Western beauty standards of thinness (5, 14, 15).

Acculturation as a complex and interdisciplinary phenomenon has been defined in multiple ways, all implying meeting of cultures and the subsequent changes in individuals or groups (16). Psychological acculturation refers to the changes an individual experiences as a result of culture change while adjusting to a new dominant culture [Graves, 1967 as cited in Ref. (17)]. One of the prominent acculturation models introduced by Berry and his colleagues is a fourfold model of acculturation proposing four acculturation strategies that individuals might apply when exposed to culture change: assimilation – preference in adopting and maintaining only new cultural identify, separation – preference in maintaining only original cultural identity, integration – preference in both maintaining original and adopting new cultural identities, and marginalization – no interest in maintaining/adopting either cultural identity (16, 18–20).

Migration-related psychological distress and mental health vulnerability of immigrants and refugees have been recognized by an abundance of research prompting researchers to propose that culture change and adopting to Western lifestyles posed certain risks for psychological well-being among diverse populations (21–24). Empirical evidence suggests that once individuals are subjected to acculturation, engagement in both cultures leads to better outcomes compared to the engagement in one culture only, while engagement in neither culture has been linked with the poorest health outcomes (18, 20, 25).

The link between culture change and disordered eating has attracted researchers’ interest towards the end of the twentieth century as EDs started emerging in non-Western countries, including Eastern Europe, and among immigrant/minority populations (8, 26–29). Cases of EDs in some parts of the world, e.g., the island of Curacao and South America, appeared very low primarily affecting elite groups exposed to North American and European influence (14, 27, 30), which further strengthened the hypothesis linking EDs with acculturation to Western culture and values.

While several researchers suggested that eating disturbances might take place in the process of adapting to a new culture (31, 32), some have linked increased ED symptomatology with initial years of immigration and lower levels of acculturation to a mainstream culture (33–36), while others identified higher risks at later stages of immigration and higher levels of acculturation (37–40). Similarly, some studies comparing various immigrant and nonimmigrant groups identified higher ED susceptibility among westernized immigrants (35, 39, 41–43), whereas others have found no associations between culture change and disordered eating (10, 44–47).

Nevertheless, the ways these studies defined and measured acculturation varied significantly (some using proxy measures such as length of residence in a new country) and many of them did not sufficiently examine acculturation variables relevant to the well-being outcomes (48–50). As the process of acculturation is multifaceted, the impact of a variety of factors (e.g., acculturative stress, internalizations of Western cultural values, acculturation variables) on one’s well-being tends to be accumulative and the individual influence of each is hard to determine. Thus, more studies are needed encompassing diverse populations to advance the knowledge on the associations between immigration, acculturation and eating patterns, and to examine the unique ways of responding to culture change by each group.

Located on the crossroads of Eastern Europe and Western Asia, with a population of less than 4 million, Georgia is a small lower middle income country (51). After regaining independence in the 1990s, Georgia went through multiple wars and economic crises prompting large numbers of people to leave the country in search of economic prospects. With the oldest history of wine-making and a distinguished national cuisine, many cultural values of Georgia, including social interactions, revolve around eating and feasting (52–54). Overall, female beauty ideals tend to be pro-slimness. While EDs were considered non-existent in Georgia until the end of 20th century, later studies identified increased ED susceptibility (55, 56).

### The Present Study

The study examined the links between immigrating to a Western country, the strategy of acculturation established and eating patterns. For the purposes of this study, Western encompasses the UK and the USA only. Under acculturation types, four strategies of acculturation were examined – integration, assimilation, separation, and marginalization (57). Under eating patterns, dietary restriction, eating concern, weight concern, and shape concern were examined. It was hypothesized that: a) females who immigrated from Georgia would exhibit more disordered eating patterns compared to nonimmigrants, and b) among those who immigrated, the acculturation strategy of integration would be associated with lowest risk/healthiest outcomes, and strategy of marginalization would be linked with the highest risk/the least healthy outcomes. Two hypotheses were tested for which quasi-experimental cross-sectional design and correlational analysis were used.

Based on the framework of the assessment of psychological acculturation (58), the variety of situational and individual factors of acculturation (acculturation conditions) impact migrants’ home-country and new-country orientations (acculturation orientations), which, in turn, affect their psychosocial well-being (acculturation outcomes). In line with the above, the study also explored several demographic/acculturation variables and examined their links with both acculturation strategies and eating patterns. Overall, considering that our study sample was non-clinical and no cut-off values had been established for Georgian population, we refrain from making clinical judgments about EDs.

## Materials and Methods

### Participants and Procedure

In total, 506 Georgian females aged 18–55 participated in the study. Among them were 253 women residing in the UK and the USA, and 253 were residing in Georgia (control group). Inclusion criteria for both groups were: female, aged 18–55, of Georgian ethnicity, born and raised in Georgia, and first language must be Georgian. Additionally, for the immigrant/sojourner group it was necessary for a participant to be residing in UK or USA for at least 6 months, while nonimmigrant participants must not have lived abroad (short stays excluded).

The selection method used was a combination of convenience sampling and snowball sampling. Since no unified database of Georgians residing in the UK or the USA existed, it was impossible to perform probability sampling. An electronic version of the survey was created and distributed among Georgians living in the US and the UK electronically. An extensive search was performed to locate corresponding communities, groups, individuals, and forums for posting and distributing the study link. Mail-outs were sent to The Society of British Georgians, The Georgian embassy in the UK, The Society of US-Georgians. Social media was also used. A similar recruitment process was applied to control (nonimmigrant) group: electronic version of survey was created and distributed among participants electronically. Nonimmigrant group survey did not include acculturation measurement. The survey was anonymous to encourage participation.

Data was first collected from immigrant/sojourner group and then from nonimmigrant/control group. Composition of the latter, as a matched group, was tailored to the age distribution of the former. The second sample consisted of 345 nonimmigrants, out of which 92 entries were removed and 253 remained with matched age group distribution. Inter-group equivalence was preserved with respect to education and marital status as well (see **Table 1**).

**Table 1:** Descriptive data of participant demographics.

Group	Age (18–55)		Highest education obtained			Marital status				Body Mass Index		
	Mean	SD	Higher	Incomplete uni-vocational	High school or less	Single	Married	Divorced	Widow	Mean	SD	Percentage per category
Immigrant (N = 253)	41	8.31	224	23	6	47	159	36	11	24.12 range 30.37	4.71	3.6% < 18.5 61%-norm 23.1%-overweight 11.9%-obese
Nonimmigrant (N = 253)	41	8.64	234	15	3	51	158	33	11	24.94 range 29.54	5.44	6.8% < 18.5 48%-norm 27.6%-overweight 16.4%-obese

Total number of participants is 506.

### Measures

#### Demographics

Demographic variables for both immigrant and nonimmigrant groups included age, marital status, highest education achieved, employment status, height and weight. Body Mass Index (BMI) scores were calculated for all participants (see **Table 1**).

Additionally, for the immigrant group, information was gathered on a number of acculturation variables, including length of residence in the new country, age of relocating to the new country, current financial status, history of marriage with a representative of a mainstream culture (i.e., British or American), history of being undocumented (i.e., illegal immigration status), among others. These variables were regarded to have potential to influence the acculturation process and the individual’s well-being. A few of them, such as length of residence and age of relocation, have been linked with both acculturation and ED outcomes in other studies (36, 39, 59, 60).

#### Acculturation Strategies

To measure acculturation strategies, the East Asian Acculturation Measure (EAAM) (61, Georgian translation Shekriladze, I., 2015) was used. EAAM is a four-dimensional self-report measure of acculturation with 29 statements on a 7-point Likert scale (e.g., “at home I usually speak English” or “Asians (in our case Georgians) should not date non-Asians (non-Georgians)”) which measures the degrees of assimilation, separation, integration, and marginalization on corresponding four subscales. The tool consists of general statements examining one’s cultural orientations (original vs host) that can be applied to any cultural group. Higher scores indicate higher degrees of corresponding acculturation strategies. The tool has been reported to have decent psychometric properties – Cronbach’s alpha (by subscale) equals to.77/.76/.74/.85 respectively (61). The instrument was validated through Confirmatory Factor Analysis (CFA) (Structural Equation Modeling [SEM] in MPLUS software) and was slightly modified. Fit indices were as follow: χ^2^ = 690.09, df = 316, p = 0.000, RMSEA = 0.07, CFI = 0.81, TLI = 0.79. Cronbach’s alpha amounted to 0.78 for assimilation subscale, 0.73 for separation subscale, 0.64 for integration subscale, and 0.82 for marginalization subscale (62). Initially developed for the East Asian population, the tool has been used to measure the links between acculturation strategies and eating patterns (45, 63). It has also been used to measure acculturation strategies of various cultures, including Eastern European (60, 64, 65).

#### Eating Patterns

The Eating Disorder Examination Questionnaire (EDEQ) (66, 67) was selected as a widely used measure of disordered eating patterns. This tool has previously been used with the Georgian population (55). It is a 36-item self-report measure that assesses individual’s experiences within the last 28 days with answers on a 7-point scale from 0 to 6, in which 0 corresponds to never/no day and 6 corresponds to every day (e.g., “Have you been deliberately trying to limit the amount of food you eat to influence your shape or weight?”, “have you tried to exclude from your diet any foods that you like in order to influence your shape or weight?”). It generates scores on four subscales – dietary restraint, eating concern, shape concern and weight concern – and a global score representing the mean of all subscale scores. Higher scores indicate more disordered eating psychopathology. The measure has shown good psychometric properties (66–68). Suggested clinical cut-off for the EDEQ global score is 2.3 (69).

### Statistical Analyses

Data was analyzed using the statistical package IBM SPSS version 21.00. First, data was assessed for normality and for outliers. Several extraneous variables were controlled for, including BMI. Bivariate correlations were performed to explore the links between demographic variables (e.g., lengths of residence, age of relocation to a new country) and EDEQ/EAAM scores. Mean EDEQ scores were compared between immigrant groups based on immigration status and the history of marriage with the representative of a host culture (i.e., British or American). Mean EDEQ scores of immigrant and nonimmigrant groups were compared. Correlational and regression analyses were performed between immigrant acculturation strategies and EDEQ scores. Collinearity was checked between the acculturation strategies prior to conducting multiple regression analyses. In line with broader research examining the links between culture change and eating patterns, probability level of 0.05 was used in all statistical tests of significance. Nevertheless, we adjusted p-values using False Discovery Rate (FDR) method producing more conservative significance threshold at q = 0.01. Thus, we decided to deem probability levels of p < 0.05 marginal.

## Results

### Participant Demographics and Their Links With Acculturation and Eating Outcomes

The immigrant group mostly consisted of adult females who moved to a Western country in their late twenties and had been residing there for about a decade. Fewer than 18% were under 30 years old and only about 20% of participants resided in a new country for the period of up to 5 years (10% – for the period of up to 3 years), whereas 80% had been living there for the period of 6+ years. More than a quarter of women reported being married to a British/American man, while one fifth reported experience of being undocumented (see **Table 2**). Nearly half (47.3%) of the participants reported having normal/average financial status.

**Table 2:** Demographics of immigrant group.

Variables	Mean	Median	SD	Minimum	Maximum	N
Age of relocation	29.43	29	7.47	10	54	
Length of residence	11.56	12	6.30	0.6	25	
Body Mass Index	24.12	23.24	4.71			
Resides in UK						105
Resides in USA						148
Married to British/American						67 (26.5%)
Undocumented						50 (20%)

Total number of participants is 253.

Length of residence was found to have a statistically significant positive correlation with integration (r = 0.26, p = 0.000) and a statistically significant negative correlation with separation (r = −0.20, p = 0.001). Age of relocation was significantly negatively correlated with both assimilation (r = −0.20, p = 0.002) and integration (r = −0.24, p = 0.000). None of these variables, however, had any statistical significance in terms of ED outcomes. Therefore, they were not further analyzed as covariates.

Out of the categorical variables, it was speculated that history of being undocumented might have a negative impact with one’s integration and well-being outcomes, whereas history of being married to a representative of mainstream culture might be linked with more favorable outcomes.

Analysis of variance (ANOVA) was used to examine differences with respect to acculturation strategies, eating patterns and BMI of immigrant women with a history of being undocumented and those without such a history. Comparisons showed no significant differences with respect to acculturation strategies or eating patterns (although the tendency was for all mean EDEQ scores to be higher for women with the history of being undocumented); however, the mean BMI of women with the history of illegal immigration status appeared markedly higher (M = 25.86, SD = 4.59) than of those with no such history (M = 23.69; SD = 4.87; F (1, 249) = 8.59, p = 0.004, η² = 0.03).

Furthermore, ANOVA was used to compare acculturation and EDEQ outcomes of immigrant women with the history of being married to British/American and those without such a history. The comparison yielded between-group differences in some EDEQ outcomes, as well as assimilation, separation and integration scores (see **Table 3**). More specifically, women who had a history of marriage with the representative of mainstream culture, showed marginally lower (i.e., healthier) EDEQ weight concern and global scores than those with no such history. Furthermore, they scored significantly lower on the EAAM separation and had higher integration and significantly higher assimilation outcomes compared to women with no such history. In other words, women with a history of being married to British/American, showed somewhat more favorable eating patterns and higher host culture orientation.

**Table 3:** Links between the history of marriage with British/American, acculturation, and EDEQ outcomes.

	Married to local		Not married to local		*F (215)*	*η²*
	*M*	*SD*	*M*	*SD*		
Food restriction	1.57	1.55	2.00	1.58	3.61	0.01
Eating concern	0.55	0.90	0.85	1.10	3.84	0.01
Shape concern	2.12	1.48	2.50	1.73	2.57	0.01
Weight concern	1.72	1.43	2.21	1.65	4.70^†^	0.01
Global score	1.49	1.11	1.89	1.30	4.97^†^	0.01
Assimilation	3.48	1.22	2.86	1.26	12.25***	0.04
Separation	2.56	0.88	3.38	1.24	24.36***	0.08
Integration	5.68	0.97	5.26	1.19	6.89**	0.02
Marginalization	2.22	1.08	2.38	1.18	0.953	0.00

***p < 0.001, **p < 0.01, ^†^p < 0.05.

### Immigration and Eating Patterns

We expected EDEQ scores to be higher among immigrant/sojourner population compared to nonimmigrant group due to the experience of immigration to Western countries. Eating patterns were measured by 5 scores – dietary restriction subscale, eating concern subscale, shape concern subscale, weight concern subscale, and global score.

Even though we do not consider it appropriate to apply the recommended cut-off value of 2.3 of EDEQ global score to our sample, we still checked how many participants in each group scored higher just for the purposes of shedding light on the distribution of extreme eating patterns. Findings showed that EDEQ global scores of 33.2% (84) of immigrants and 31.6% (80) of nonimmigrants exceeded the recommended cut-off value with rather similar distribution of the data in the two groups.

Comparisons of the two groups were performed by one-way between-groups Multivariate Analysis of Variance (MANOVA), which showed notable difference between the EDEQ restriction scores (F (1, 504) = 6.27, p = 0.013, partial η2 = 0.012) only and marginal difference between EDEQ eating concern scores (F (1, 504) = 4.03, p = 0.045, partial η2 = 0.008), with the former being higher among immigrants, and the latter being higher among nonimmigrants. It should be noted though that nonimmigrant group had marginally higher mean BMI (24.94) than immigrant group (24.12). To ensure the equivalence of the groups, BMI as a confounding variable was controlled. Before checking links between BMI and EDEQ scores, the linearity of their relationship was checked. BMI appeared significantly correlated with all the EDEQ outcomes (r = 0.27, r = 0.39, r = 0.55, r = 0.50, r = 0.51, n = 504, p < 0.001 in all cases) and thus linearity was observed.

Next, a multivariate analysis of covariance (MANCOVA) with BMI as a covariate was performed, which showed that the marginal difference between the two groups with respect to eating concern disappeared (it appeared to be explained by BMI), whereas the difference with respect to dietary restriction became stronger (see **Table 4**). In other words, as immigrant group with lower BMI produced a higher restriction score than nonimmigrant group with higher BMI, the deference between the groups’ restriction scores became more important. Thus, for Georgian women, moving to Western countries for a prolonged period increased dietary restriction. Hence, in the context of EDEQ outcomes, moving to the West appeared to affect only one out of five EDEQ subscales: restriction of food intake. The global EDEQ scores of immigrant and nonimmigrant groups, however, were almost identical.

**Table 4:** EDEQ scores of immigrant and nonimmigrant groups.

EDEQ scores	Group ID	M	SD	N	MANCOVA	Partial η^2^
					*F*	
EDEQ	Immigrant	1.89	1.59	253	7.53**	0.02
Restriction score	Nonimmigrant	1.55	1.50	253		
EDEQ	Immigrant	0.78	1.06	253	2.45	0.01
Eating concern score	Nonimmigrant	0.97	1.10	253		
EDEQ	Immigrant	2.40	1.68	253	0.43	0.00
Shape concern score	Nonimmigrant	2.50	1.61	253		
EDEQ	Immigrant	2.08	1.61	253	0.15	0.00
Weight concern score	Nonimmigrant	2.03	1.56	253		
EDEQ	Immigrant	1.79	1.27	253	0.06	0.00
Global score	Nonimmigrant	1.76	1.19	253		

**p < 0.01.

Since EDEQ cut-off values had not yet been established for Georgian women, no clinical judgments were made about disordered eating. Although the norms of our study groups (both immigrant and nonimmigrant) were markedly higher than UK (66) and Australian (70) community norms, we did not deem appropriate to make comparisons neither from cultural, nor from an age perspective.

### Acculturation and Eating Patterns

Among immigrant/sojourner population, we expected the strategy of acculturation with the mainstream culture to be linked with eating patterns: namely, integration associated with the lowest EDEQ scores (most favorable patterns) and marginalization with highest EDEQ scores (the least favorable outcomes).

### Correlational Analyses

Bivariate correlations between the strategies of acculturation and EDEQ scores were performed through controlling BMI as a confounding variable. Prior to correlational analyses, the linearity of the relationship between BMI and EDEQ scores was checked. Immigrant group BMI appeared significantly correlated with all the EDEQ outcomes (r = 0.22, r = 0.37, r = 0.55, r = 0.49, r = 0.49, n = 251, p < 0.01 in all cases), and thus linear relationship was observed.

Correlational analyses showed that marginalization and separation appeared to have strong statistically significant positive correlations with eating concern, shape concern, weight concern, and global scores. Integration appeared to have marginal negative correlations with eating concern and shape concern outcomes (see **Table 5**). No correlations were identified between EDEQ restriction concern and any acculturation strategy. The strategy of assimilation was not linked with any EDEQ score either.

**Table 5:** Correlations between EAAM acculturation scores and EDEQ Outcomes.

EDEQ outcomes	Marginalization	Separation	Integration	Assimilation
EDEQ eating concern	0.30***	0.30***	−0.14^†^	0.06
EDEQ shape concern	0.26***	0.27***	−0.16^†^	−0.04
EDEQ weight concern	0.24***	0.28***	−0.07	0.01
EDEQ global	0.26***	0.27***	0.30	0.02

***p < 0.001, ^†^p < 0.05; N = 253.

### Regression Analyses

Finally, multiple regression analyses were conducted to examine the extent to which the different acculturation strategies may predict unhealthier eating in our sample. For each of four EDEQ outcome, a stepwise selection method was used to enter BMI and acculturation scores into the model. The results of regression analysis showed that separation and marginalization strategies were linked with EDEQ eating concern, shape concern, weight concern and global scores. Out of the two, marginalization was linked mildly, while separation showed stronger links (see **Table 6**). No associations were identified between integration and eating and shape concern scores.

**Table 6:** Stepwise regression analysis results of EDEQ outcomes and EAAM acculturation scores.

Model	Eating concern	*β*	*T*
Model	Shape concern	*β*	*T*
Model	Weight concern	*β*	*T*
Model	Global EDEQ score	*β*	*T*
1	BMI	.37	6.34***
2	BMI	.35	6.32***
	Separation	.22	3.46**
	Marginalization	.15	2.38*
1	BMI	.55	10.64***
2	BMI	.53	10.67***
	Separation	.16	2.84**
	Marginalization	.14	2.39*
1	BMI	.49	8.89***
2	BMI	.46	8.79***
	Separation	.21	3.58***
	Marginalization	.13	2.08*
1	BMI	.49	8.87***
2	BMI	.46	8.83***
	Separation	.20	3.35**
	Marginalization	.15	2.46*

***p < 0.001, **p < 0.01, *p < 0.05.

Thus, regression analysis further showed that two strategies of acculturation, separation and marginalization — can be considered predictors of higher EDEQ scores on four out of five EDEQ subscales, with separation having strongest predicting value.

### Summary of Results

**Figure 1:**
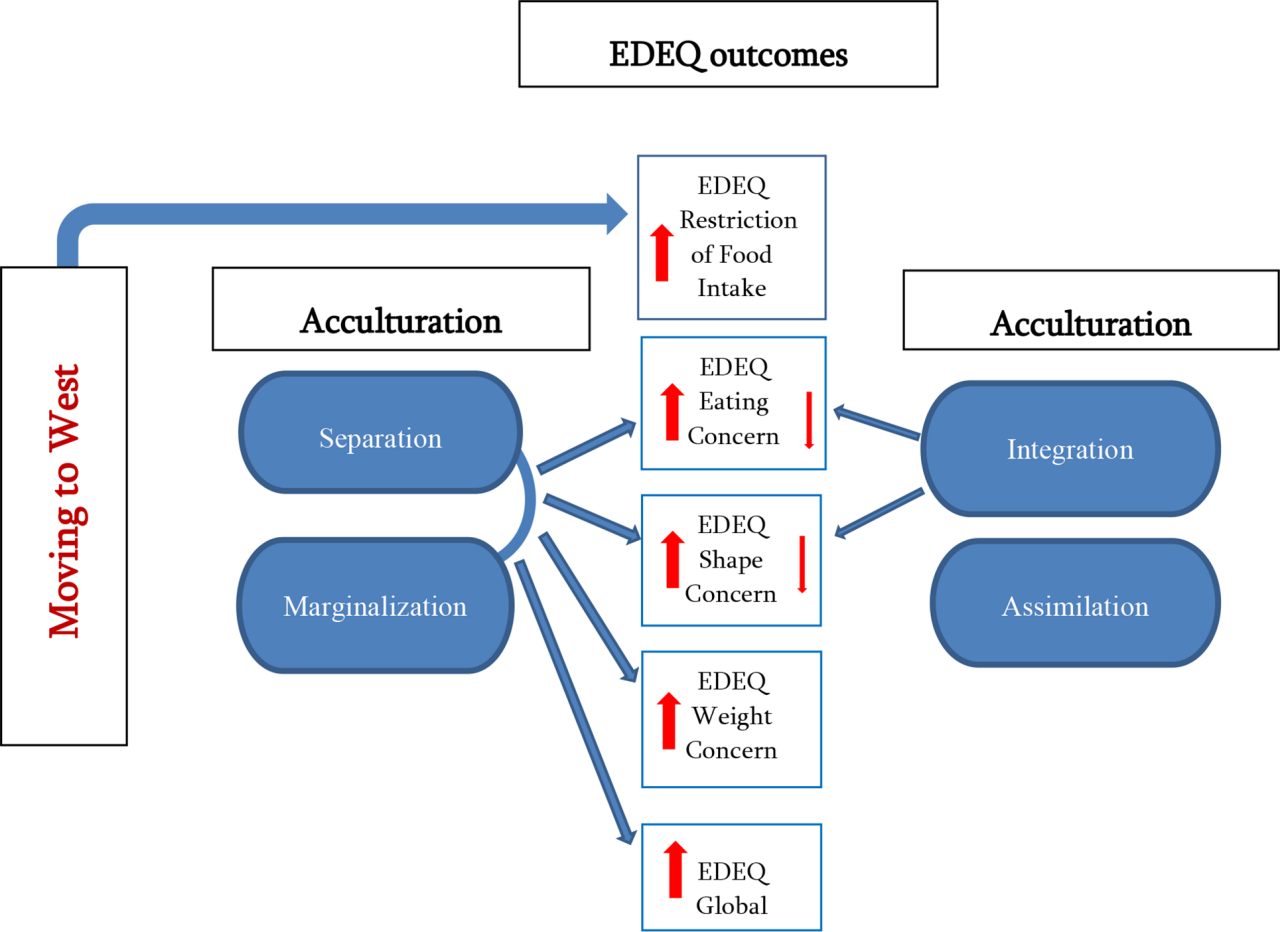
Links between moving to West, acculturation strategies and five eating pattern indicators (EDEQ outcomes). **Moving to West** linked only with restriction of food intake; **Separation** and **marginalization** considerably linked with eating concern, shape concern, weight concern and global EDEQ outcomes; **Integration** mildly linked with eating concern and shape concern outcomes; **Assimilation** not linked with any EDEQ outcomes.

In summary, the findings on the links between immigration, acculturation strategies and eating patterns of Georgian immigrants showed that: (a) moving to a Western country appeared to increase restriction of food intake; (b) while living in a Western country, acculturation strategies of separation and marginalization were associated with higher eating concern, shape concern, weight concern and global scores of EDEQ (**Figure 1**); in addition, history of marriage with a representative of host culture was linked with higher host culture orientation and lower EDEQ scores.

## Discussion

### Acculturation Conditions

Length of residence, education, cultural distance and expectations were reported to be important factors in adjustment by various prominent acculturation researchers (71–73). Consistent with above evidence, in a present study, both length of residence and early relocation age have been correlated with higher levels of integration. They were not linked with eating patterns.

Surprisingly, history of being undocumented (illegal status) as an acculturation condition generally impeding one’s integration into society of settlement, was not linked with acculturation strategies or poorer scores in EDEQ. It was, however, associated with higher BMI. On the other hand, history of marriage with a representative of mainstream Western culture, as an acculturation variable evidently facilitating one’s integration, was associated with higher host culture orientation and somewhat healthier eating outcomes.

It needs to be recognized that the cases of the USA and the UK, as societies of settlement, might be different from other Western countries in many ways (from one another as well) and, therefore, while formulating interpretations, it is important to avoid generalizations to all “Western” world, especially when the characteristics of the society of settlement have not been duly explored.

### Culture Change and Eating Patterns

The results of the present study on the links between immigration, acculturation and eating patterns can generate significant new insights. Putting together the findings of comparisons of immigrant vs nonimmigrant groups, on the one hand, and within-immigrant group (based on their acculturation strategies or other variables), on the other, allows us to see a bigger picture.

Our findings allowed us to conclude that higher dietary restriction (the only difference between immigrant and nonimmigrant groups out of five EDEQ indicators showing statistical importance) among immigrants could be attributed to moving to the West. It is questionable, however, whether increased restriction is a manifestation of eating pathology (as it did not affect EDEQ global score). Furthermore, based on the findings, eating concern, shape concern, and weight concern, can go up if individual applies separation or marginalization strategies of acculturation. In other words, restriction (behavioral by nature) is increased for all immigrants/sojourners irrespective of their acculturation strategies, whereas eating concern, shape concern and weight concern (all cognitive by nature) tend to go up when individual strategy of acculturation implies lack of integration in a host culture (low host culture orientation) (see **Figure 1**). The importance of integration into mainstream society with respect to healthy eating is further strengthened by additional findings linking the history of marriage with a representative of host culture with higher host culture orientation and more favorable eating outcomes.

Based on the findings, we might speculate that Western context puts more restrains on food intake as opposed to Georgian context, thereby contributing to increased dietary restriction for all. On the other hand, taking into consideration the fact that the nonimmigrant sample had a higher number of participants with out-of-normal BMI, we might also envisage elevated restriction among immigrants as a means of ensuring more normal BMI (presumably due to the higher emphasis on healthy eating more prevalent in the West than in Georgia). Nevertheless, BMI could not explain the differences in EDEQ scores between immigrant and nonimmigrant groups; neither could it explain the links between EDEQ and acculturation strategies, as it was controlled as a covariate in both cases. Thus, the findings allow us to pinpoint the higher predicting value of a lack of integration in the society of settlement in developing higher eating, shape, and weight concerns among Georgian sample. In other words, it is not moving to the West per se that can contribute to unhealthier eating, but maintaining the low host culture orientation in a new country of residence (**Figure 1**).

### Immigration

Overall, our findings were consistent with the evidence supporting minor or no differences between ED vulnerability of immigrant and nonimmigrant populations (44–47). Nevertheless, the reasons for generating such findings might be sample specific. First of all, multiple studies have shown that most people integrate after a certain period of time (20, 74, 75), while acculturative stress and culture shock are highest during the initial periods of firsthand contact with another culture [(57, 59, 76); Oberg, as cited in Ref. (71)]. In addition, it is recognized that forced migration and uprooting complicates psychological well-being of migrants (22), while voluntary migration in search of a better future is associated with favorable adjustment outcomes (Richmond, as cited in 71). Considering above, our sample is in an advantageous position: it consisted of relatively veteran (6+ years) immigrants, as average length of residence was 11.5 years, who by the time of our study had presumably adjusted one way or another. Besides, for this sample, relocation to Western countries was a preferred choice in search of economic and life prospects. Had there been different composition of participant length of residence and different circumstances of relocation, the outcomes might have differed as well.

Some research evidence suggests that the higher the distance between home and host cultures, the stronger the acculturative stress and the poorer the well-being outcomes (25, 77, 78). Culture encompasses what constitutes culinary and mealtime traditions as well as esthetic ideals. Even though, on average, Georgians eat more than Europeans, Georgian mealtime sequence (breakfast, lunch, and dinner) as well as most culinary ingredients are similar to European and slimness is admired in females. Therefore, as certain similarities and slender beauty ideals are present, the impact of cultural clash caused by internalization of Western habits and beauty standards may not be as dramatic as in case of some other groups examined in ED research. Its relative cultural proximity to contemporary Western world may play part in lowered risks of disordered eating.

In addition, Georgians, as invisible minorities, publicly may be perceived as representatives of mainstream culture, which gives them a considerable advantage over some other immigrant groups that have been popularly studied in the field of acculturation and EDs (e.g., Chinese, Africans, East Asians). Studies show that being a visible minority is associated with higher perceived discrimination and poorer adjustment outcomes (24, 79), including ED patterns (80).

Thus, to summarize, our findings suggest that prolonged residence in Western countries is associated with increased dietary restriction among Georgian women. This, however, may not be necessarily a manifestation of disordered eating. Variety of sample-specific factors, such as, longer length of residence, voluntary immigration, being white, and Christian, and less dramatic distance between cultures are speculated to have played protective functions making the process of acculturation less stressful. These variables have been linked with higher levels of integration and better well-being outcomes in other studies [Richmond as cited in Refs. (25, 71, 81)].

### Acculturation

Our findings were consistent with studies linking unhealthier eating with lower levels of acculturation to the host culture (33–36). Namely, in case of Georgians, a weak mainstream-culture orientation, as a common feature of separation and marginalization strategies, appeared to predict higher EDEQ scores, thereby making strong mainstream-culture orientation a protective factor (**Figure 1**). These findings also corresponded to evidence of a meta-analysis on acculturation and mental health showing that orientation to mainstream culture was linked with favorable adjustment outcomes (82, 83) and lower levels of depressive and anxiety symptoms (84). Likewise, in 2014 study on 7,000 Canadian immigrants, Berry and Hou (75) found that both integration and assimilation strategies were linked with the highest scores of life satisfaction, whereas separation and marginalization were associated with significantly lower scores.

Nevertheless, perhaps the most important finding of the study was that, unlike Berry’s model, separation was associated with higher EDEQ outcomes than marginalization, representing the strongest predictor of elevated eating, weight, and shape concerns (and EDEQ global score), whereas assimilation was not at all linked with disordered eating. While marginalization is generally regarded the least favorable strategy of acculturation (20, 25, 84, 85), the reasons why in the present study it was outweighed by separation might be context specific.

While separation strategy is a natural reaction on culture change at the initial stages of immigration, one might speculate that after years of living in another country, it might be indicative of serious internal or external difficulties of adjustment. In contrast to separation, marginalization as a condition that implies cultural identity confusion, might be more natural after spending a substantial period away from one’s home as opposed to early stages of relocation. Moreover, in the long term, assimilation may turn into another conventional and authentic acculturation strategy and, as demonstrated by our findings, linked with favorable outcomes. Therefore, it might be argued that acculturation is a dynamic process (25, 86) and it is time and stage-specific which strategy of acculturation is most appropriate for an individual or group.

Furthermore, as some researchers argue, different acculturation strategies might work in different contexts (82, 87) as context-specific characteristics may determine which acculturation strategy is most relevant. For instance, separation might be natural and even externally driven option for representatives of cultures/nations with big communities in the societies of settlement, such as the US Latin American populations, who have huge Spanish-speaking communities. In contrast, Georgians are few and scattered in the US and UK and do not necessarily enjoy strong ties with their ethnic population. Thus, for our sample with average length of stay of 11.5 years and 80% of participants living in a new country for 6+ years, to carry on a separation strategy after years of relocation may indeed entail considerable adjustment difficulties, isolation and inability to live in the present (e.g., excessive reminiscing about old times in the country of origin and/or excessive dreaming about returning to the home country), all contributing to the least favorable EDEQ outcomes.

### Summary

To conclude, the findings on the links between immigration, acculturation and eating patterns of Georgian women showed that dietary restriction is increased for all immigrants/sojourners regardless of their acculturation strategies, whereas eating concern, shape concern, and weight concern go up when individual’s strategy of acculturation implies lack of integration in a host culture (low host culture orientation). Hence, our evidence suggested that a weak host culture orientation may be considered as a risk factor of unhealthier eating, thereby reiterating critical value of immigrant integration into mainstream culture with respect to dietary health and potentially better overall well-being. The results also supported that acculturation is a very multifaceted process affected by a number of acculturation conditions (e.g., arrival age, length of residence, etc.) among which, history of marriage with the representative of a host culture was linked with higher host culture orientation and more favorable eating behaviors. Since a variety of acculturation conditions are related with how people adjust to new cultures and how their psychosocial functioning proceeds, determining the impact of culture change should entail a comprehensive approach addressing acculturation attitudes/behaviors, well-being outcomes, and major acculturation variables.

## Limitations and Future Directions

The main limitation of the study was sampling bias due to the impossibility of probability-sample that limits the generalizability of findings. Our study could not be free of limitations associated with self-report e-surveys. Despite friendly instructions, inaccurate completions are possible.

In addition, fewer than 18% of the sample were under 30 years old and only 20% of our sample appeared to be residing in a new country for 5 or less years. Thus, the findings cannot be generalized to a younger age group during which typically EDs emerge, or to a newly relocated. Therefore, it would be valuable for future studies to target younger and newly relocated groups of Georgian immigrants/sojourners and examine their response to culture change and acculturative stress.

Furthermore, the study could not explore potential comorbidities and the variety of important acculturation variables potentially affecting well-being outcomes, such as, command of a language of a host country, ties with original culture and characteristics of the society of settlement (e.g., perceived discrimination, food industries, etc.). Future direction of research might look at the differences between the countries of the so-called Western world, including British and American, examining the above characteristics.

Another important future research direction might be looking more closely at immigrant and nonimmigrant samples with regards to eating patterns and elaborating on why behavioral (dietary restriction) pattern appeared universal for immigrant group in terms of being elevated and the cognitive ones (eating concern, shape concern, weight concern) – subjected to conditionality (only in cases of lower mainstream culture orientation).

Finally, our findings suggested that for Georgian women who have been residing in a Western country for 6+ years having high host culture orientation is important with respect to healthier eating patterns. As culture change is a very multifaceted process, duly examining variety of factors (e.g., circumstances of relocation, cultural distance between original and host cultures, social support, etc.) that shape the adjustment and well-being outcomes, including eating patterns, seems essential for seeing a bigger picture.

## Data Availability

The datasets generated for this study are available on request to the corresponding author.

## Ethics Statement

Study ethics (03-11563/15) permission was obtained from Ilia State University Ethics Committee, in line with the Declaration of Helsinki, providing for the rights of participants, including anonymity of individuals and their data protection.

## Author Contributions

IS carried out the study, performed the analysis and drafted the manuscript. NJ took part in the data processing and analysis. KT was the lead supervisor of the study helping with the recruitment, training, design and write-up, and editing the manuscript. All authors provided feedback and contributed to the final manuscript.

## Funding

This study was partially supported by Shota Rustaveli National Science Foundation of Georgia (grant number: YS/17/15-815/15). KT would like to acknowledge Prof Tracey Wade and the Norman Munn Distinguished Visiting Scholar Award 2017/2018 from Flinders University, South Australia.

## Conflict of Interest Statement

The authors declare that the research was conducted in the absence of any commercial or financial relationships that could be construed as a potential conflict of interest.

## Abbreviations

ANOVA, Analysis of Variance; EAAM, East Asian Acculturation Measure; BMI, Body Mass Index; CFA, Confirmatory Factor Analysis; ED, Eating Disorder; EDEQ, Eating Disorder Examination Questionnaire; FDR, False Discovery Rate; MANCOVA, Multivariate Analysis of Covariance; MANOVA, Multivariate Analysis of Variance; SEM, Structural Equation Modeling.
